# A Narrative Review on Rice Proteins: Current Scenario and Food Industrial Application

**DOI:** 10.3390/polym14153003

**Published:** 2022-07-25

**Authors:** Gopika Jayaprakash, Aarti Bains, Prince Chawla, Melinda Fogarasi, Szabolcs Fogarasi

**Affiliations:** 1 Department of Food Technology and Nutrition, Lovely Professional University, Phagwara 144411, Punjab, India; gopikanalupankil@gmail.com; 2 Department of Microbiology, Lovely Professional University, Phagwara 144411, Punjab, India; aarti05888@gmail.com; 3 Department of Food Engineering, University of Agricultural Sciences and Veterinary Medicine of Cluj Napoca, Calea Mănăstur 3–5, 400372 Cluj-Napoca, Romania; melinda.fogarasi@usamvcluj.ro; 4 Faculty of Chemistry and Chemical Engineering, Department of Chemical Engineering, Babeş-Bolyai University, 11 Arany Janos Street, 400028 Cluj-Napoca, Romania; 5 Interdisciplinary Research Institute on Bio-Nano-Sciences, Babeş-Bolyai University, 42 Treboniu Laurian Street, 400271 Cluj-Napoca, Romania

**Keywords:** rice protein, amino acid, physicochemical characteristics, health benefits, cytotoxicity, food applications

## Abstract

Rice, *Oryza sativa*, is the major staple food that provides a larger share of dietary energy for more of the population than other cereal crops. Moreover, rice has a significant amount of protein including four different fractions such as prolamin, glutelin, globulin, and albumin with different solubility characteristics. However, these proteins exhibit a higher amino acid profile, so they are nutritionally important and possess several functional properties. Compared with many other cereal grains, rice protein is hypoallergic due to the absence of gluten, and therefore it is used to formulate food for infants and gluten-allergic people. Furthermore, the availability makes rice an easily accessible protein source and it exhibits several activities in the human body which discernibly affect total health. Because of these advantages, food industries are currently focusing on the effective application of rice protein as an alternative to animal-based and gluten-containing protein by overcoming limiting factors, such as poor solubility. Hence, it is important to gain an in-depth understanding of the rice protein to expand its application so, the underlined concept of this review is to give a current summary of rice protein, a detailed discussion of the chemistry of rice protein, and extraction techniques, and its functional properties. Furthermore, the impact of rice protein on human health and the current application of rice protein is also mentioned.

## 1. Introduction

Cereal grains are one of the salient foods in the human diet and widely consumed cereals include maize, wheat, barley, and rice while sorghum, oat, millets, and rye are consumed at a lower level. These grains provide the required nutrients for mankind such as carbohydrates, protein, fiber, crude fat, essential fatty acid, minerals, and vitamins for healthy living [1]. A cereal grain-based breakfast provides numerous health benefits, including lower postprandial blood glucose levels, improved insulin responses, increased satiety, and reduced long-term weight gain [2]. Moreover, cereal grain consists of three parts including the embryo, pericarp, and endosperm. Among them nutrient-rich part of a grain is the endosperm, however, the composition varies from species to species [3]. The germ portion is rich in antioxidants, vitamin B and E, phytochemicals, and lipids whereas bran, the fiber-rich outer layer contains B vitamins, trace minerals, and bioactive phytochemicals such as phenolic compounds (including lignans, alkylresorcinols, and phenolic acids), phytosterols, and carotenoids [4]. After the starch, the protein is the second-largest component in the endosperm, which represents approximately 10–12% of dry weight [5]. Among all cereals, rice (*Oryza sativa)* is almost exclusively used for human food, and it is the staple crop for more than half of the world’s population as it provides required nutrients for healthy living and primarily it is used in low- and middle-income countries which account for 20% of human calorie food intake. Due to its high nutritional quality and digestibility rice is considered the queen of cereals [6,7]. Rice has protein as its second dominant (6–7%) major nutrient. Based on the Osborn solubility classification, rice protein has four fractions namely, glutelins, albumins, globulins, and prolamins and their solubility are in alkali, water, salt, and alcohol, respectively [8]. The proportion of albumin, globulin, glutelin and prolamin has been reported to be 5–10, 7–17, 75–81, and 3–6%, in brown rice, 4–6, 6–13, 79–83, and 2–7%, in milled rice, and 24–43, 13–36, 22–45 and 1–5% in rice bran, respectively [9]. Therefore, the dominant protein in rice is glutelin and the salt-soluble prolamins are minor, which is generally the major storage protein in other cereals except for oats [10]. The net protein utilization of rice is the highest among cereal grains [9]. The protein-dense portion in the rice grain is known as dense deposits of protein bodies (PB). There are two types of PBs, PB I and PB II with different structures and compositions. These PBs are known for their amino acid profile comprising essential amino acids like threonine, leucine, and phenylalanine along with sulfur-rich amino acids like methionine and cysteine [11]. Furthermore, rice has an unbalanced amino acid composition with higher glutamine (15–31%), proline (12–14%), leucine (7–14%), and alanine (4–11%), and lower nutritionally important amino acids such as tryptophan (0.2–1.0%), methionine (1.3–2.9%), histidine (1.8–2.2%), and lysine (1.4–3.3%) content [12]. In addition, arginine, isoleucine, methionine, glycine, serine, tyrosine, and valine are also found in rice [13]. Amino acids, concentrated in the germ, are glutamic acid, gamma-aminobutyric acids, aspartic acid, leucine, lysine, proline, threonine, and tryptophan [14]. The protein isolates from rice are highly nutritious and similar to casein and soy protein isolates. In addition, rice protein isolates have been highly recommended for infants and the elderly due to their nutritional quality, digestibility, and hypo-allergenicity, so rice protein is considered an alternative source of protein over animal-based protein [15]. Despite this, lower solubility in water hinders the potential application of rice protein commercially. However, the industries and research communities possess an interest in developing more preferable processes for the extraction, enrichment, purification, and functionalization of rice protein. As a result, the traditional alkaline, enzymatic, and physical extraction techniques are modified to obtain more extract of protein regardless of their low solubility. On other hand, the storage protein in the rice endosperm appears more easily extractable. However, inducing denaturation or aggregation due to these processes makes protein difficult to extract [16].

Meanwhile, the possibility of using rice by-products as a food ingredient is increasing nowadays. Protein obtained from the rice bran is used in several food industries such as bread, breakfast cereals, protein supplements, beverages, and even meat and sausages. As rice protein contains glutelin instead of gluten, it is suitable for producing gluten-free products which can be used to feed gluten-sensitive people [17]. Furthermore, rice protein isolates, the byproduct of glucose and starch manufacturing, are used as a protein source with 60% of protein with a reasonable amino acid composition. Despite its low solubility rate rice protein isolates are used widely as an infant food ingredient. The application of rice protein in food varies by functional properties (forming properties, emulsifying properties, water absorption properties, oil absorption properties) [18]. The limiting factor for the preparation of value-added rice proteins is their low solubility; in water, it is less than 2% and the pH range of 4–7. As discussed above the extensive aggregation and crosslinking through hydrophobic interaction and disulfide bonds result in the insoluble precipitate. Therefore, the current application of rice protein is limited. In the last few decades, chemical and biochemical techniques have been adopted to enhance solubility but these bring out a reduction in the functionality and nutritional properties of rice protein. Recently, researchers have been focusing on other methods like protein-protein interaction, conjugations, and encapsulation to improve rice protein’s functional properties including gelation, emulsification, and primarily solubility to make their nutritional quality available for mankind [18].

In this article, the structural characteristics, as well as the composition of the rice grain and rice grain protein, were investigated. The structural characteristics including molecular weight, particle size distribution, surface hydrophobicity, secondary structure, amino acid composition, and chemical bonds of rice protein were evaluated. Furthermore, the functional properties, pharmaceutical importance, and the recent application and status of protein derived from rice grain were preliminarily discussed.

## 2. Protein Extraction

**Figure 1:**
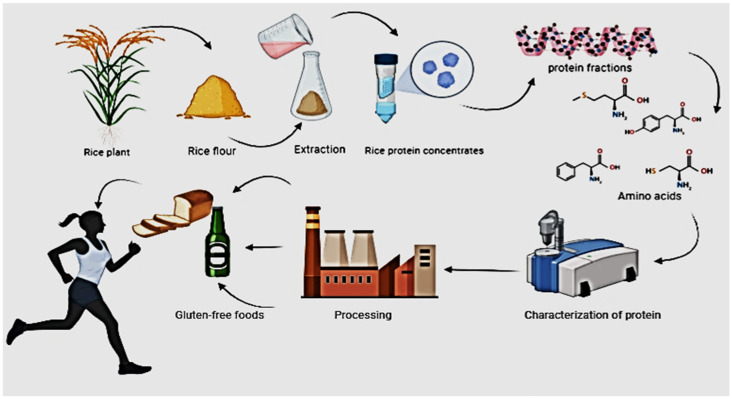
A simplified representation of the life cycle of rice protein from the rice plant to the human body.

Plant-derived protein has an important role as an alternative protein source over animal protein. Compared with animal proteins, these proteins are less expensive and abundant in supply. Increased utilization of plant-based protein completely depends on the extraction technology based on raw materials [19]. In former times, little interest was given to rice protein processing because of relatively low protein content and lesser commercial opportunities. Conventional as well as novel methods have been utilized to extract protein from rice with a minimal effect and a high rate of yield. In this regard, the role of rice protein in human health is briefly described in Figure 1. As mentioned above the functional and physicochemical properties of these proteins are extremely dependent on the method used for extraction [20]. Normally, wet and dry protein techniques are used for extraction purposes such as two-phase system, subcritical water, and reverse micelles extraction. There are more extraction methods such as conventional and non-conventional cell disruptive methods including aqua-based, solvent, detergent, alkali and enzyme assisted, microwave, homogenization, pulse-filed, high pressure, and sonication to extract protein from various plant parts. The conventional method is subjected to changes in extraction time, pH, temperature, and solvent used and it is liable to lower yields due to the degradation of proteins whereas the non-conventional method uses non-thermal green technology to reduce degradation and thus results in higher yield, nutritional and functional properties [21].

### 2.1. Chemical Extraction

Chemical extraction of protein involves the use of different solvents such as alkali, water, acids, and other organic solvents [5]. This technique is useful to isolate protein with minimum loss and maximum yield. Despite this, the efficiency of this method is dependent on the nature of the protein sample. Solvent used extraction comprises mainly three steps such as defatting, extraction, and precipitation of protein from a raw sample. The first step involves the treatment of the sample with fat-extracting solvents (n-hexane, petroleum ether, n-pentane) which is then followed by extraction of protein using a cold or hot water-based aqueous extraction technique with the addition of salts, ionic or non-ionic detergents. For organic solvent extraction ethanol, methanol, or buffers can be used. Finally, extracted protein is concentrated by a precipitation technique using any precipitation agents including acetone, methanol, ethanol, trichloroacetic acid, ammonium sulfate, citric acid, hydrochloric acid, or by isoelectric point precipitation. After centrifugation, a protein isolate is obtained without any impurities and non-protein compounds [21].

#### 2.1.1. Solvent Extraction

For isolation of protein aqueous extraction is commonly used because of the purity of the end product, i.e., protein concentrates. This kind of extraction focuses on the solubility and stability of the extracted protein. In the solvent extraction method alcohols such as ethanol, butanol, and acetone are used to extract hydrophobic, polar, or non-polar protein from the respective protein source [19]. An aqueous-ethanol (20–90%) assisted extraction of protein from rice flour using centrifugation at a temperature of 50–100 °C and same with the addition of salt of NaOH shows a variation in the percentage of extracted protein due to the solubility, folding of the protein, and denaturation of protein and the higher amount is obtained for the concentration of 70% (*v*/*v*) and at 70 °C [20].

#### 2.1.2. Alkali Extraction

The most widely used conventional method for extraction purposes is alkali extraction. In this technique, the protein is exposed to alkali such as NaOH and KOH for dissolving the protein by breaking the disulfide. In the food industry, this treatment of rice flour is generally applied to facilitate the removal of the endosperm proteins. As the name suggests, an alkaline condition is required for the extraction of the glutenin fraction. This is due to the ionization of acidic and neutral amino acids occurring at the basic pH. Therefore, alkaline extraction gives a significant yield of protein. In addition to the pH, temperature has a crucial role in protein yield [22]. Several previous studies showed that approximately 97% of the protein was extracted using diluted NaOH or KOH from rice flour. The ultrafiltration or isoelectric precipitation using higher pH of 11 at 40 °C resulted in 71 and 86% of protein yield, respectively, with superior functional properties. Moreover, an increase in solubility was observed at a pH of 10 and 12 in the result proposed by Singh et al. [20]. In addition, the wet milling of soaked rice in 0.3–0.5% sodium hydroxide for more than 24 h dissolves more proteins [23].

On other hand, the use of alkaline extractions mainly alters the native structure of a protein in extreme pH conditions with an increase in both functional and bioactive properties. Moreover, this treatment may also promote many modification reactions, which lead to changes in the chemical, physical, and nutritional properties of food proteins by the destruction of cysteine and lysine amino acids [22,24]. When the protein comes into contact with an alkali, it will undergo denaturation, causing the unfolding of the alpha-helical region which then forms a β-sheet and thus reduces the amino acid interactions. This reduction is due to the conversion of amide groups into carboxyl groups. An increase in alkalinity results in an increase in β-sheet content and a decrease in α-helical and β-turn content [25,26]. In rice, the lysine acts as the limiting amino acid so the exact condition is to be selected to prevent the loss of nutritive value of rice protein [22]. Therefore, the protein prepared by alkaline extraction has higher digestibility and bioavailability. However, this technique also has some drawbacks, as alkaline conditions could lead to denaturation and hydrolysis of proteins, and non-protein components may co-precipitate with proteins during increased extraction, thus lowering the purity of the resultant protein; due to the higher pH range darkening occurs due to increased Maillard reactions, and the formation of toxic compounds such as lysinoalanine is also possible [7].

#### 2.1.3. Enzymatic Extraction

Enzymatic extraction is considered a method using an enzyme to extract protein and it is a commercial method for food application purposes without any environmental pollution which also fully recovers protein from the food processing waste. Various enzymes are used to dissolve specific components to extract the bonded protein easily. It is common to use enzymes such as pectinase and carbohydrase to digest the cell wall; similarly, protease is used to unfold the polysaccharide matrix to release and fractionate the larger protein into smaller and soluble proteins. In the case of cereals, both pectinase and carbohydrase (cellulase, pectinase, arabinose, β-glucanase, xylanase, hemicellulase) are used to extract protein [19]. Furthermore, protein isolates with 90% of protein content are obtained by using heat-resistant starch hydrolyzing enzymes. Since starch represents the major constituent of rice endosperm, enzymes such as α-amylase, glucoamylase, and pullulanase, which solubilize starch, are often used to separate proteins in rice flour. The use of carbohydrate hydrolyzing enzymes such as cellulase or a combination of cellulose and hemicellulase in addition to the α-Amylase enriches the protein in the residue [11]. This method of separation captivates attention due to the lower pH conditions thus the physicochemical and functional properties of the isolated protein remain practically unchanged [23].

### 2.2. Physical Extraction

Physical extraction is the method that induces less modification in extracted protein. Moreover, it is more economic and easier to adapt to various industrial environments. The most frequently used physical methods to extract protein from rice include high-speed blending, sonication, hydrothermal cooking, freeze-thaw, micro fluidization, and the use of subcritical water. About 15% of protein can be extracted by using sonication and it is the highest quantity of protein to be extracted by using a physical method. The action of enzymes such as a-amylase and protease will increase the extraction ability. Both micro fluidization and density-based separation of milled rice contribute a yield of 82% with a purity of 44% which will increase further to 88% if treated with amylase and glucoamylase enzymes. The resultant protein is known for its in vitro digestibility but shows less solubility in the pH range of 6–10 compared with alkaline extracted protein. Unusually, the protein isolates obtained through the HTC method exhibit higher surface hydrophobicity, sulfhydryl, and disulfide bond contents and show superior emulsifying properties, compared with alkali-extracted protein. Extraction of protein by using the subcritical condition of the water is an emerging method that uses water at a temperature between 100 and 374 °C and provides a higher pressure to maintain its liquid state. Temperature and dielectric constant are inversely proportional when the temperature increases from ambient 250 °C, its relative dielectric constant decreases from around 80 to values close to 27, which is close to ethanol at ambient temperature therefore subcritical water can dissolve hydrophobic material and due to high dissociation constant, it can catalyze chemical reactions like degradation and hydrolysis, therefore, subcritical water can hydrolyses protein without an external catalyst [7,22].

#### Ultrasonic-Assisted Alkali Extraction

**Figure 2:**
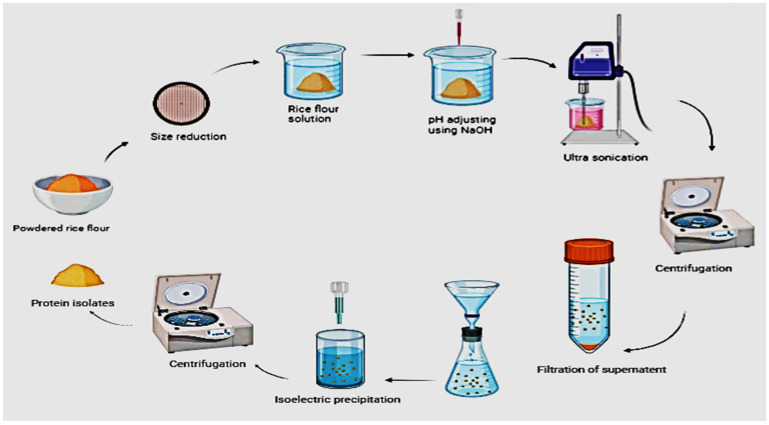
Schematic representation of ultrasonic-assisted alkali extraction of rice protein.

Ultrasonic-assisted alkali extraction on the non-thermal physical processing technology. This process involves the use of both alkali and ultrasound to extract protein. Some of the steps are similar to traditional alkali extraction, which includes, centrifugation for the removal of insoluble material, filtration, and isoelectric precipitation as shown in Figure 2. The steady flow of fluid generated by ultrasound causes the rapid production and collapsing of gas bubbles which results in high shear strength and mechanical energy over the cell wall or membrane which disturbs the cell by leaving cavitation, which facilitates the extraction process through the disruption of cell walls as well as the membrane [19,25]. This facilitates the penetration of solvent used for extraction purposes by improving its mass transfer and releasing the intercellular protein into the solvent. The pH, power, time interval, frequency, solvent ratio, and intensity of ultrasound collectively affect the efficiency of this technique. Several advantages can be noticed for this method, as a non-thermal process it gives low-temperature treatment to avoid the denaturation of protein and loss of functionality. Moreover, the extraction time and amount of solvent needed are considerably less compared with other conventional methods [26]. In addition, the preferred condition for rice protein extraction is 750 W power output with a frequency of 25 kHz at 25 °C for 30 min for the maximum extraction of 23% and prolonged exposure of the protein to the ultrasound tends to cause denaturation [27].

## 3. Chemistry of Rice Protein

### 3.1. Fractions of Rice Protein

Albumin: The water-soluble protein fraction of rice is albumin, with a yield of eight peptides upon hydrolysis. The sufficient net charge and the lack of any extensive disulfide crosslinking or aggregation give its solubilizing property. This fraction is heat liable, denaturation occurs at 75.7 and 73 °C, and coagulation may occur, possessing a lower number of disulfide bonds, and readily digestible and absorbable [28]. It comprises 2–6% of total seed protein, 24–37% of bran proteins, and 4–8% of the endosperm. This fraction contains proteins that have mainly 10–200 KDa, among them, 16 KDa and 60 KDa glycoprotein predominate. In rice bran albumin generally contains proteins less than 100 kDa molecular weight [29].

Globulin: Globulin fraction is salt-soluble due to its net electrical charge [7], the second most abundant rice seed protein, containing proteins rich in cysteine and methionine but lysine constitutes a lesser amount. It is about 15–36% of the storage proteins of the bran. Generally, it shows a molecular weight of 10–15 KDa. The reduction of disulfide bonds in globular fraction proteins results in the formation of polypeptides of 16 kDa γ-globulin and 21 kDa α-globulin [30].

Glutelin: Glutelin, the primary storage protein in rice, is extensively aggregated, disulfide-bonded, glycosylated, and difficult to solubilize. It is considered alkali-soluble (pH > 10) and acid-soluble (pH < 3). Rice glutelins are composed of high-molecular-weight proteins ranging from 45 to 150 kDa. Divided into two subunits, with MW of 30–40 KDa which are acidic (α) and 19–23 KDa are basic (β) in nature [29,30]. The amino-acid composition of glutelin is not so distinct from that of total rice protein because it is the most abundant protein. The glutelin fraction in rice endosperm contains several polypeptides of 57 kDa, 34–37 kDa, 25 kDa, 21_23 kDa, 16 kDa, 14 kDa, and 57-kDa. The rice glutelin protein consists of two subunits of 30–35 kDa (acidic α-subunit) and 19–25 kDa (basic β-subunit) linked by disulfide bonds. Glutelins are poorly soluble in water but are readily solubilized in alkali (pH > 10) and acidic conditions (pH < 4). Approximately 11–27% glutelin is present in bran and 66–78% is present in endosperm mainly in PB II. Just behind albumin, glutelin contains a good amount of lysine. Glutelin is said to be present at around 75–81%, 79–83%, and 22–45%, in brown rice, milled rice, and rice bran, respectively [11].

Prolamin: Prolamins are also called reserve proteins because they are found in the endosperm area of the grain. They are deficient in essential amino acids but have non-essential amino acids [31]. Rice prolamins soluble in aqueous ethanol (60–70%) are about 4% of the bran, the minor fraction in rice seed constitutes 2.6–3.3%. Glutamine/glutamic acid, alanine, glycine, and arginine-rich polypeptides of prolamin fraction are between 12 and 17 kDa but they are low in lysine [6]. Leucine, valine, and glutamine are the amino acids found in prolamin so is considered the rich source of these amino acids but lacks lysine. On the other hand, the fractions 16 kDa and 10 kDa are rich in sulfur-containing amino acids, such as cysteine and methionine. The low solubility of prolamin in water is due to the high content of acid amides and low content of polar amino acids. They are hydrophobic and localized in PB I [11].

### 3.2. Molecular Weights of Rice Protein Fractions

**Figure 3:**
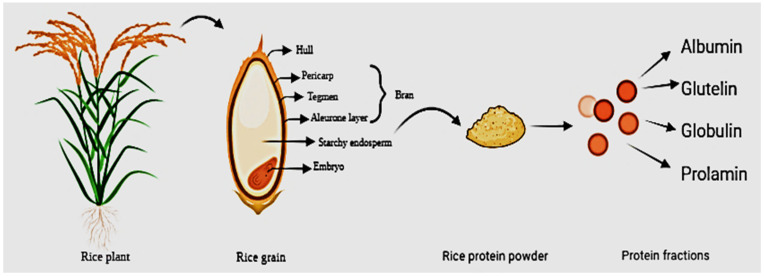
Morphology and internal structure of rice kernel with grain endosperm protein fractions.

The molecular weight of rice protein fraction is discussed as, for albumin; 30–45 KDa, for globulin; 20–66 KDa, glutelin; 10–66 KDa and prolamin; 10–53 KDa. On the other hand, rice bran proteins made up of fractions like albumin, globulin, glutelin, and prolamin, as shown in Figure 3, come in the range of 10–100 KDa, 10–150 KDa, 33–150 KDa, and 25–100 kDa, respectively. Rice albumin is highly heterogeneous [31] because it contains protein with MW 10 to 200 kDa. Noticeably the pre-dominant weights are 16 kDa protein and 60 kDa glycoprotein. Rice is subdivided into groups concerning its molecular weight (MW) ranging from 13 to 110 KDa [28]. The prolamin fraction generally contains proteins in the molecular weight range of 10–17 kDa. Its relative molecular mass lies from 33 to 150 kDa with the majority concentrated around 105 kDa [32].

### 3.3. Isoelectric Point

The isoelectric point of albumin is considered as pH 4.1 and 6.4 (for 60 KDa glycoprotein fraction) and enthalpy of 2.88 J/g. Globulin is pH 4.3, 5.85–7.27, and 7.9, and glutelin is reported as pH 4.8, 5.7–6.8, and 8.0–8.7. Prolamin is hydrophobic and localized in PB I so its IP (isoelectric point) ranges from pH 6.0–6.5 [30].

### 3.4. Molecular Structure

Rice proteins have three structures namely α-helix, β-sheet, β-turn, and random coil. Among them, α-helix has a dense ordered structure, whereas β-sheet and β-turn have a relatively extended ordered structure, while random coil has a disordered structure [18]. The secondary structure of proteins has a different ability to adapt to the surrounding environment. The β-sheet is more sensitive to environmental changes and processing conditions than other structures [32]. When considering the rice protein fractions, rice globulin has a similar structure to oat globulin. The presence of a random coil structure, an antiparallel chain of an intramolecular β-sheet structure that is absent in albumin gives the solubility characteristics of globulin. It is protein-rich in cysteine and methionine, sulphur-containing amino acids, and a low level of lysine. Its protein stabilization property is dependent on the covalent and non-covalent forces and its hydrophobic and di-sulfide interaction influences the aggregation behavior of this fraction [11].

### 3.5. Amino Acid Composition

The nutritional quality of a protein is closely related to its essential amino acid contents and the bioavailability of these amino acids. The amino acid composition will reflect the chemical and physical properties of food [33]. Compared with other cereals rice proteins are easily digestible and have higher biological value with higher protein efficiency quotients. Moreover, rice proteins are a rich source of amino acids which is a balanced and complete proposition compared with wheat and corn. It is due to the higher contents of lysine and sulphur-containing amino acids. Generally, 18 amino acids including 8 essential amino acids such as isoleucine, leucine, lysine, methionine, phenylalanine, threonine, tryptophan, and valine are found in rice (Table 1). Normally, rice protein contains histidine in a range of 1.0–3.8%, threonine; 3.15–4.43%, valine; 5.0–7.31%, methionine; 0.8–1.77%, phenylalanine; 1.18–5.81%, isoleucine; 3.60–5.35%, leucine; 6.90–8.82%, and lysine, 1.3–5.10% [13]. Prolamins have been known for having the lowest content of lysine, which is the first limiting amino acid among cereal proteins, whereas the highest content of lysine is found in rice albumin then glutelin, and last globulin. Compared with brown and milled rice, rice bran is rich in albumin, so it displays a higher content of lysine. Furthermore, histidine and threonine are also present in albumin, but in the case of prolamin, isoleucine, leucine, and phenylalanine are the commonly present amino acids. The globulin fraction has the highest content of cysteine and methionine, the sulfur-containing amino acids while prolamin has the lowest. Therefore, albumin has been estimated to have the highest biological value (BV) and prolamin has the lowest [7].

Rice protein isolates contain a larger amount of methionine and phenylalanine than casein and leucine, and threonine than the soy protein isolate. Compared with fenugreek protein isolates, rice protein isolate is rich in histidine, methionine, and phenylalanine. Therefore, the combined use of other plant proteins with rice protein may result in a complete protein source [37]. Besides this, various processing techniques like high protein flour production, acid hydrolysis, and others destroy a wide range of amino acids such as isoleucine, methionine, threonine, tryptophan, and valine mostly due to oxidation. Therefore, the processing of rice protein into various foods requires more care [13,38].

### 3.6. Surface Hydrophobicity

The surface hydrophobicity of protein plays an important role in the determination of stability, and functional properties of the protein. It is a type of noncovalent interaction between the ligands which is correlated to the foaming, emulsifying, and gelatinization properties and thus to the food application of the protein [34,39]. Rice protein is highly hydrophobic due to the presence of hydrophobic amino acids. Oxidation may cause an increase in hydrophobicity because of the exposure of more hydrophobic groups. Denaturation, expansion of peptide chains, dissociation of subunits, and unfolding of protein also lead to the exposure of both buried hydrophobic groups and side-chain groups of hydrophobic aliphatic and aromatic amino acids which are embedded inside, thus enhancing the hydrophobicity of the resultant structure [40,41]. Moreover, temperature changes seriously affect hydrophobicity. At higher temperatures, the degree of transformation increases and causes conversion from ordered structure to the disordered structure which results in the flexible and loose molecular structure of the protein. This change in structure enhances the appearance of hydrophobic residues on the surface thus increasing the surface hydrophobicity [42].

## 4. Characterization of Rice Protein

### 4.1. Fourier-Transform Infrared Spectroscopy (FTIR)

FTIR spectroscopy is a powerful technique used to detect characteristic vibrational absorption bands of molecular groups as well as to provide the changes in groups and surroundings. In terms of protein, it gives the structure and compounds that are present in a protein’s secondary structure. Moreover, FTIR has a wide range of uses and can be used to study the relationship between the structure and functions of proteins and detailed analysis of the impact of point mutation, and substrate binding on the structure and stability of protein [43]. In addition to these, it is applicable for examining protein structures in different physical states, more accurately the denatured state. The infrared spectrum helps to check up on the group and structure that are present in an element matrix. According to various reports, FTIR is used in a range of 4000 to 400 cm^−1^ region with a resolution of 4 cm^−1^ but most of the secondary structure of the protein, the amide I band, can be seen in the range of 1600 and 1700 cm^−1^. The amide I band is nothing but the stretching of C=O of peptide bonds. Several authors have documented the structure and behavior of the protein. In their studies the fractions of amide I band normally shown, the β-sheet structure at 1615–1638 cm^−1^ region, random coil at 1638–1645 cm^−1^, α-helix at 1645–1662 cm^−1^, and β-turn structure at 1662–1682 cm^−1^ [28,44]. At the same time, Piotrowicz confirmed that the spectra which reveal the protein concentrates lay at 1667.59 cm^−1^, and they confirm the presence of β-sheet formed by the reversion of 180° of the polypeptide chain. It is formed by comprising four amino acid residues common to the presence of asparagine, glycine, cysteine, proline, and tyrosine, these amino acids are folded together and stabilized by hydrogen bonds [35]. Zhao studied the amide I band using the same region of 1600–1700 cm^−1^. In this study, he observed that rice protein has a densely ordered α-helix and extended ordered β-sheet and β-turn, and finally, a disordered random coil. He also stated that the content of these four structures changes in accordance with storage time and temperature. From that finding, it was observed that β-sheet is more sensitive to environmental changes [45]. In a previous study, alkali-treated rice protein and its structural changes using FTIR found that alkali-treated rice protein showed less random coil and β-sheet whereas a large amount of β-turn, and α-helix means an increase of ordered structure and decrease of disordered structures. This is mainly due to the degradation of protein into small molecular peptides [46]. Apart from that a strong absorption of spectra was seen in the range of 3440 and 3070 cm^−1^ which is related to the vibration of hydroxy and N-H bond. Commonly, amide band is observed for secondary structure analysis. In that case, a range between 1615–1638 cm^−1^ was for the b-sheet structure, 1638–1645 cm^−1^ was the random coil, and 1645–1662 cm^−1^ was for the a-helix structure, 1662–1682 cm^−1^ was β-turn structure. Most of the time Gaussian curve fitting was used to study the secondary structure distribution [39].

### 4.2. Differential Scanning Calorimetry (DSC)

Differential scanning calorimetry is a technique used to analyze the thermal properties of powder samples like protein which precisely reveal the structural and conformational changes. By analyzing the DSC-thermograms we obtain both the peak temperature (i.e., Tp) or the denaturation temperature (i.e., TD, the peak of the denaturation curve) and enthalpy of denaturation (i.e., ∆H) [15]. The peak temperature gives knowledge of proteins’ thermal stability whereas the enthalpy gives information about the hydrophobic/hydrophilic interactions and compactness of viewed protein or proportion of undenatured proteins or the extent of ordered protein structure. There have been numerous studies of rice protein, which have used a temperature range of 5 to 100 °C at a rate of 5 min^−1^ and found that protein denaturation might occur at a temperature of (Tp) 72.8 °C to 82.2 °C and goes up to 85.6 °C (endosperm protein) and enthalpy of 0.15 Jg^−1^ [36,40].

### 4.3. X-ray Diffraction (XRD)

X-ray diffraction analysis provides information related to the crystalline nature of the protein. An X-ray diffractometer is a tool that facilitates observation in this method. Previous reports suggested some commonly used conditions, such as voltage 44 kV; current 30 mA; Cu-kα radiation with 0.1542 nm wavelength. From the obtained diffractogram the nature of protein can be found using the 2 θ scale. Scanning range 4–40 with the rate of 5/min at room temperature can be used [47]. Similarly, Singh et al. [20] observed the crystallinity of protein using 40 kV voltage and 20 mA current with a Cu-kα radiation of 1.79 Å. The following observation was done based on the diffractogram obtained at an angle of 2 θ from 0–90° with a scanning speed of 3°/min and found that at 22° rice protein exhibited crystallinity with a characteristic sharp peak.

### 4.4. Scanning Electron Microscopy (SEM)

Scanning electron microscopy is an analytical tool used to analyze the morphology of powders using secondary electron signal imaging. About 15 kV of voltage is commonly used to analyze the powdered protein under a scanning electron microscope. Moreover, the gold coating is also present in the analysis. Normally, rice protein powder has a non-uniform particle with a compact structure. Various treatments such as ultrasound pretreatment and sonication induce changes in particle size as well as the structure of the protein [45]. Similarly, a continuous space structure was observed by Hou et al. [40] for rice protein, and the surface morphology changes with treatment applied to the protein, i.e., alkali treatment causes the changes from honeycomb structure to the continuous sheet then a smooth continuous surface with an increase in concentration respectively.

## 5. Techno Functional Properties of Rice Protein

### 5.1. Solubility

The solubility of protein depends on the balance between hydrophilic and hydrophobic regions of the protein molecule and the exposed amino acid on its surface. The higher solubility represents the exposure of hydrophilic amino acids which interact well with water molecules that decreases the hydrophobicity of rice protein [48]. Protein solubility is directly proportional to temperature, i.e., it increases with an increase in temperature. This relationship is effectively used in alkaline extraction by facilitating higher temperatures to extract small oligomers and lower temperature allows the release of large aggregates from rice protein [30]. Moreover, the pH changes also affect the solubility, protein extraction from milled rice can be increased significantly with alkalinity and acidity. For example, a minimum solubility for commercial rice protein is shown at pH 5 whereas at pH 2 it is higher than 55%. The lower aqueous solubility of intact rice protein is mainly due to the presence of glutelin, the major protein fraction in rice protein. Substantial aggregation, hydrophobic interactions, and crosslinking through disulfide bonds are the cause of the low water solubility of glutelin [49]. Increased dissociation of glutelin aggregates will promote solubility in acidic or alkaline pH. Native glutelin extract from milled rice flour has a solubility of 8% at pH 5 and it can be increased up to 16% and 30% by changing the pH to 2 and 8 respectively. Reducing the molecular weight of rice protein molecules and enhancing the number of ionizable groups by subjecting intact protein to enzymatic hydrolysis helps to increase the solubility of rice protein hydrolysates as a result of protein hydrolysis [47].

### 5.2. Buffering Capacity and Viscosity

Buffering capacity of rice protein ingredients is considerably higher than that of dairy protein in a pH range of approximately 3–4 so it is more resistant to acidification or alkalinization [37]. Sulfhydryl and disulfide bonds are crucial in defining protein functions, such as gel formation and film formation [50]. In fresh rice the free sulfhydryl bonds present ~0.72 mM/g and ~2.6 mM/g disulfide bonds [51]. A process like oxidation causes the conversion of sulfhydryl to disulfide bonds and the spatial structure of protein becomes loose, both resulting in low rice viscosity [52].

### 5.3. Emulsifying Property

**Figure 4:**
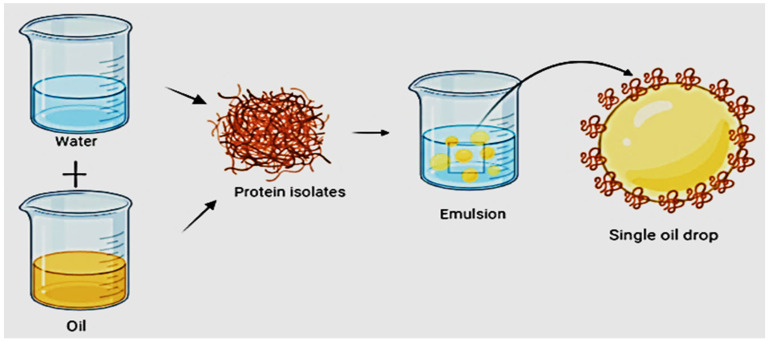
The symbolic presentation of emulsifying behavior of rice protein with the newly formed dispersed particle in the protein-mediated emulsion.

“Emulsifying property” is a term used to define emulsifying activity index, emulsifying capacity, and emulsifying stability. Rice protein has a weak emulsifying activity because of low water solubility and high disulfide bonds [53]. Rice protein emulsifying properties depend on protein surface charge, the hydrophobicity, hydrophilicity, and solubility of the protein. Among these hydrophobicity and aggregations are the dominant ones as well as pH, which has an important role in emulsifying properties directly proportional to each other because alkaline and acidic pH improve the solubility of protein which results in shattered disulfide bonds [46]. In addition, Figure 4 gives a schematic representation of the interaction of rice protein with oil molecules in an emulsion formation. Decreased hydrophobicity of protein ends in low emulsifying property due to less oil and protein interaction. The emulsifying property greatly depends on the oil and protein interactions so an increase in the surface hydrophobicity enhances the property as well [11].

### 5.4. Water and Oil Binding Capacity

Rice proteins are both hydrophilic and hydrophobic so they can easily interact with water and oil. It is considered the ability of the protein to imbibe and hold water in its matrix. This interacting ability of proteins gives them special properties such as improved freshness, mouthfeel, enhanced flavor, and retention in a food product [44]. Oil absorption happens during various processing steps such as heating, which tends to unfold protein structure and reveals more hydrophobic groups hence entrapping more oil physically. Intact rice protein and its hydrolysates have less oil absorption capacity compared with hydrolyzed protein because it breaks protein chains, hence exposing buried internal hydrophobic groups. The oil absorption capacity of brown rice and white rice protein is 2.93 mL/g and 2.56 mL/g, respectively, whereas the water holding capacity is about 2.81 g/g [23].

### 5.5. Foaming Capacity

The foaming capacity and foaming stability of protein are associated with the ability to reduce the surface tension at the water-air interface and are closely related to the structure of the protein [54]. The forming capacity is mainly affected by penetration, transportation, and rearrangements of the molecule under the air-water surface. High temperature denatures the protein structure and this degradation and disaggregation in proteins cause an increase in foaming capacity and foaming stability. During denaturation, protein unfolds and lets the particles accumulate which reflects in higher foaming properties [55]. Moreover, processes like freeze milling and alkali treatment cause an increase in both foaming capacity and foaming stability. Dramatic changes in foaming characteristics may be due to changes in insolubility and rapid unfolding of protein which forms a cohesive layer around gas or air droplets. Furthermore, the hydrophobic nature of rice protein shows a positive correlation with foaming capacity because the hydrophobic patches present on the protein surface interact with aqueous molecules [56].

### 5.6. Polyphenolic Compound’s Binding Properties

Phenolic compounds are natural antioxidants and possess at least one aromatic ring with one or more hydroxyl groups. This structural characteristic gives the antioxidant ability [56]. Protein and polyphenols interact with each other by reacting with side-chain amino groups of peptides thus forming protein crosslinks and the resultant product exhibit improves functional qualities [57,58]. Protein polyphenol interaction or conjugation is achieved through both reversible and irreversible methods, this interaction improves protein functionalities like surface hydrophobicity and emulsifying property so it can be used to design functional ingredients [59,60]. Phenols are responsible for the interaction because they have an amphiphilic structure that contains both hydrophilic hydroxyl group and hydrophobic aromatic nucleus. Therefore, the reactions result in the formation of either covalent or non-covalent binding with the reactive biomolecule. Hydroxyl groups are capable of donating or accepting hydrogen hence forming hydrogen bonds with the carbonyl and hydroxy amino groups of proteins respectively [61]. Phenolic interaction can change the structure and functional properties of the protein. Source of protein, pH, surface properties of the protein, amino acid composition, number of hydroxyl groups, isoelectric points, temperature, the type of structure, and the type of interaction are the factors that affect protein-phenolic interaction [62,63]. The variation in pH denatures the protein’s native structure to expose the hydrophobic groups. In acidic pH (~pH 3) rice protein exposes its protein core region and unfolds partially to provide a binding site for hydroxyl group and aromatic groups in phenols. These binding characteristics destruct secondary structures as well as the functional groups of proteins. Therefore, it alters the hydrophobic-hydrophilic balance and interfacial behaviors [59,64]. Hydrophobic interaction occurs between side chains of amino acids and benzene rings of the interacted polyphenol compounds, resulting in the formation of the hydrogen bond. The electrostatic force also imparts interaction, but it is considered a secondary force. Several hydroxyl groups on the benzene ring can also affect the binding behavior of proteins with phenols [61].

## 6. Application of Rice Seed Protein in Food Industries

Rice protein is considered a superior source of protein to other plant-based protein sources because of its nutritional excellence also used as a novel food ingredient due to various advantageous functional properties [56]. The protein obtained from rice is low cost and high quality thus it is becoming an alternative to the existing protein supplements. As discussed above rice protein is obtained by different extraction methods, certain modifications help to extract the protein with minimum deterioration compared with the practiced one which elevates the solubility of different fractions in the desired food. Rice protein is used in food formulation because of its foaming and emulsifying properties [65].

### 6.1. Gluten-Free or Hypoallergic Product

A gluten-free diet is preferred for those patients with celiac disease, throughout their life due to the damage caused by the gluten (mixture of prolamins) to the villi of their small intestine thus resulting in lower absorption of nutrients. Cereal grains are considered the major source of gluten except for rice so other cereals such as wheat, rye, barley, triticale, and some oats add a considerable amount of prolamins to the diet. Replacing wheat in many foods especially baked foods because of its unique rheological, textural, and sensory characteristics of gluten is a serious challenge [66]. Nowadays different formulations of protein, starch, and fibers are being used to produce gluten-free foods. The protein of these formulations is mainly obtained from rice, which is rich in amino acids, lysine, and hypo-allergenicity (glutelin instead of gluten). Higher digestibility, bland taste, and colorless properties also ensure a preference for rice protein over other plant-based proteins. Several studies have given evidence for the feasibility of utilizing rice protein as a substitute for allergic proteins. Therefore, rice protein is used in the formation of gluten-free baked products for gluten-allergic people and also in making infant formulations for the nourishment of infants having allergic problems with milk protein. Moreover, rice is nowadays used to produce gluten-free beverages such as beer. Rice was used only as an adjunct in past years whereas all-rice malt beer is now being formulated to meet consumer requirements [67].

### 6.2. Protein-Based Meat Products

Food insecurity is a serious issue that all developing countries face nowadays. Approximately 60% of the world population is suffering from a deficiency of animal-based protein due to the unavailability and high cost of meat and meat products. Enhancing the use of economical non-meat protein sources helps to reduce the occurrence of protein deficiency diseases. Rice protein, mainly rice protein isolates, can be used as an extender, plant-based protein additives, in meat products to enhance the water holding and textural properties which reflects in improved volume or yield, quality of product, and sensory properties [65]. Moreover, the use of a rice protein-rich source of lysine used as an extender will also provide essential amino acids to consumers. Several studies show that, by using rice protein, the moisture content of the end product increases in the range of 53 to 72–60.09%, whereas the pH of the product will decrease with an increase in protein concentration (5.62–6.34). The incorporation results in improved gelation, swelling, and viscosity of products such as sausage. Rice protein isolates have low moisture content which results in improved texture quality (up to 5.56) and also increases the hardness due to the imbalance in the emulsion process. An improved tenderness is common in extended products, while it shows a high cooking loss [68]. The main advantage of using rice protein in meat is that it has strong antioxidant properties, so it helps to improve the shelf life of the meat products by reducing lipid oxidation. Furthermore, the protein, fat content, and redness of meat also increase [65].

### 6.3. Edible Films and Coating

Edible films are useful as a carrier of nutrients as well as flavors and antioxidants. It has other preservative properties which enhance the shelf life of the product and also add value to the product. Due to high tensile strength, protein-based edible films are used to reduce the use of plastic-based packages. Compared with lipid-based films, proteinaceous films are strong and impermeable to oxygen due to their tight packaging and ordered hydrogen-bonded network. Protein film formation is done by denaturation by acid, alkali, or other treatments, and occurs with the help of acid, alkali, or other treatments resulting in the formation of hydrogen, ionic, hydrophobic, and covalent bonds forming a protein matrix. Protein molecules interact mainly through di-sulfide, hydrophobic, and hydrogen bonds. Improvements in flexibility can be achieved by adding low molecular weight plasticizers [69].

Normally, rice protein is a readily available, edible, non-toxic, and biodegradable raw material, however, due to low molecular weight and self-crosslinking rice protein are incapable of forming a film. Previous studies showed that film formulation using rice protein is possible by using 70% protein solution (dry basis), glycerol as a plasticizer, and a pH of about 9.5 or 3.0. The change in pH has an impact on the color and transparency of film, i.e., a higher pH produced a darker, intense reddish-yellow tan color, and lower pH, the extra transparent film is obtained. In addition, rice protein-based film shows good tensile strength, water vapor permeability, and less oxygen permeability. Aside from that, rice protein edible film is more biodegradable than its counterparts [70]. Moreover, the presence of propylene glycol alginate and oil provides increased water vapor resistance which creates water barrier properties in the films. High pH conditions lead to high crosslinking between amino acids which is responsible for the improved strength of these films [71].

### 6.4. Baking

Rice flour is widely used in the baking industry due to its tasteless and odorless properties. The flour plays the role of a thickening, bulking, and flavoring agent. It helps improve the rheological behavior and the end-use quality. Moreover, the richness of amino acids improves the nutritional aspects without compromising their final quality [72]. In rice, gluten is absent, so it has the advantage of making gluten-free bakery products. Rice-based biscuits, bread, cake, and buns have a potential scope because of the lack of gluten, a common allergen [68]. To produce gluten-free baked products non-glutinous rice can be used. To meet the attributes of products several additives such as viscosity improvers, and volume enhancers can be used to form a gluten-like protein network. Recently, rice flour bread was formulated by the addition of water, sugar, salt, yeast, and oil. At the same time, gluten-free pasta is also now producible depending solely on rice flour with high consumer acceptance and nutritional benefits [73].

### 6.5. Emulsifier

In this era, increased demand for plant-based natural emulsifiers has been noticed. Protein has a natural ability to form an emulsion due to the presence of both hydrophobic and hydrophilic amino acids. However, rice endosperm protein concentrates or isolates show an emulsification property, so it is used as a natural emulsifier in food products. The reduced allergen content makes rice protein a potential alternative to emulsifiers based on other plant-derived proteins such as soy, pea, and wheat. While the limited application in the food stream is due to its insolubility and instability, several treatments such as enzyme hydrolysis, freeze milling, and glycosylation help to improve the emulsifying property of rice protein effectively [74]. Therefore, rice protein isolates are now suitable for an array of food products like coffee whiteners, toppings, beverages, confectionaries, meat, and bakery products. These can be incorporated into liquid foods like milk and other drinks [75].

### 6.6. Pharmaceutical and Therapeutic Uses

Medical and therapeutic applications of rice protein are increasing day by day. Many health benefits such as effective antioxidants, antitumor properties, as well as hypertension-reducing properties, and the ability to deliver bioactive compounds are the contributors to the application. Rice protein supplements can be used as alternative infant foods because the higher lysine and threonine content helps to meet the required amino acid profile [76].

## 7. Health Benefits

Among other cereal proteins, rice protein has various health benefits. It is a low-fat source, especially with low cholesterol and heavy metal content, which helps to recover muscle strength. Moreover, it lacks gluten so it can be used to feed people who are allergic, and it is very easily digestible, so it has several applications [77,78].

### 7.1. Anti-Inflammatory and Anti-Cancer Reaction

Inflammation is the key factor behind the formation of tumors and ultimately cancer. This health issue is significant to those who consume a high-fat diet. The protein extracted from rice has a suppressive effect on proinflammatory cytokine which is related to the high carbohydrate-fat-rich diet. The consumed rice protein hydrolysate inhibits the production of proinflammatory factors and reduces further reactions [77].

Cancer is a common and life-threatening disease all over the world. Consumption of natural bioactive compounds positively influences the risk of many cancers. However, the rice-derived protein isolates seem to possess anti-cancer activity through their prolamin fraction. It is capable of inhibiting both the murine leukemia L1210 cells and the human leukemia Jurkat cell. This activity is evidence of the prolamin immune modulatory effect. Furthermore, the purified form of the same can reduce the weight of the tumor so it possesses anti-tumor immunity and can reduce the spread of leukemia without any toxicity [79].

### 7.2. Suppression of Hyperglycemia

Hyperglycemia is a major contributor to diabetes mellitus which leads to serious health issues. However, several studies suggested that rice protein possesses a potential activity against hyperglycemia. Alkali-extracted alcalase enzyme hydrolyzed rice protein suppresses hypertension by inhibiting the conversion of angiotensin I to vasoconstrictor angiotensin II which causes antihypertensive vasodilator bradykinin. This activity is due to the presence of alcalase or protamax enzyme in the protein hydrolysates produced from albumin and glutelin. This enzyme inhibits the action of angiotensin I converting enzyme. The Thr-Gln-Val-Tyr peptide sequence also possesses the same inhibiting property. The enzymatic treatment of rice albumin and globulin shows strong inhibiting activities than normally obtained globulin and prolamin hydrolysates [29].

### 7.3. Reduction of Cholesterol

The increased risk of heart disease is closely related to the cholesterol level in the blood. The effect of rice protein on cholesterol completely depends on its amino acid composition and gastrointestinal digestion. The amino acid methionine shows a hypocholesterolemic effect because this amino acid has an important role in cholesterol metabolism. Moreover, rice protein can enhance fecal steroid excretion which reflects the capacity to reduce the cholesterol level in both plasma and liver, especially the triacylglycerol levels. This is due to the properties of the protein that binds with bile acid. Alkaline and α-amylase extracted 23 KDa glutelin and 13 KDa prolamin show this property. Comparing the two, α-amylase extracted protein is more effective than the alkaline extracted protein fractions because it is more detestable and promotes fecal extraction of bile acids in the body [80].

### 7.4. Anti-Oxidative Activity

The production of plant-based antioxidant compounds has grown in importance in the last few decades. Noticeably, proteins and peptides show great activity in inhibiting oxidation reactions. Normally, enzymatically extracted proteins exhibit anti-oxidant activity. In the case of rice protein, the albumin fraction has more antioxidative activity than other fractions [81]. It can prevent oxidation of low-density lipoprotein caused by Cu ions, and surprisingly it can prevent the 16 KDa albumin from having an N-terminal amino acid sequence of Asp-His-His-Gln. Which is homologous to the sequence Asp-Ala-His-Lys of human serum albumin. Compared with albumin, globulin has less antioxidant activity, even though peptides with 670–3611 Da obtained from peptic hydrolysis show remarkable antioxidant activity. In other words, hydrophobic amino acids are more active in free-radicle scavenging activity than polar amino acids [29].

## 8. Cytotoxicity of Rice Protein

Food allergy is an emerging public health issue across the world. It is a kind of immunological response when a certain food enters the digestive tract. Generally, egg, milk, soybean, fish, wheat, and peanuts cause an allergic reaction in humans [80]. Rice is considered a non-allergic food material even though it causes negligible allergic reactions in some people such as abdominal cramping, nausea, vomiting, rhinitis, rhinoconjunctivitis, asthma, and contact urticaria, atopic dermatitis, dermatitis, and angioedema [82]. Major identified types of allergens in unpolished rice are Ory s LTP, a lipid transfer protein (LTP) of 14 KDa; Ory s aA/TI, a 16 KDa α-amylase or trypsin inhibitor; Ory s 12, profilin, particularly albumin fraction (14–16 KDa). Moreover, proteins with 26, 33, 52, 56, 63, 90, and 55 KDa molecular weight also have potential allergenicity, namely, 26 KDa as α-globulin, 90 KDa as α-glucosidase, 33 KDa as β-glyoxylase. Moreover, the remaining 52 KDa are similar to globulin protein, 63 KDa is similar to embryo globulin whereas 55 KDa represents the protein-containing disulfide isomerase [28]. The allergen 33 KDa is a novel type of plant glyoxalase I which is common in plant tissues especially maturing seeds. The α-amylase or trypsin inhibitors present in rice are responsible for the cross-allergenicity between cereals and *Poaceae* to cooked rice. Raw rice shows higher allergic potential than the processed alternative because of various processing techniques such as alkaline hydrolysis. Enzyme digestion can reduce the number of allergens from the rice [83]. Even though, some of them are heat-stable and resistant to proteolysis. Previous studies suggest that 14–16, 33, 56, and 60 KDa protein exhibit higher binding efficiency with immunoglobulin E, and IgE, and those 16, 23, 33, and 53 KDa retain their binding property even after boiling conditions. Ory s 12 profilin is common in rice seeds and contributes to allergic rhinitis and conjunctivitis. LTPs are responsible for the allergenicity of cooked rice due to its heat stability. Protein obtained through hydrolysis such as enzymatic or alkali induced and high-pressure treatment at 100–400 MPa show the actual hypoallergenic properties [28,84].

## 9. Conclusions and Future Perspective

Rice protein has a lot of potential as a nutritious, affordable, and widely available protein source. It is considered a novel food ingredient and an effective replacement for existing cereal and animal-based protein sources. The characteristic nutritional value and functional properties give a variety of applications to this plant-based protein. They have high biological value due to the presence of balanced amino acid composition, including threonine, leucine, and phenylalanine. Moreover, it is known for the sulfur-containing amino acid methionine and cysteine. Its hypoallergic nature is a useful property that is dramatically used in the production of various allergen-free food products. Furthermore, rice protein is useful in producing plant-protein-based emulsifiers and foaming agents. While rice protein has many advantages, the application of rice protein is limited due to the complexity of its structure, hydrophobic nature, covalent and noncovalent bonding, and the difference in viscosity actin as a roadblock in solubility hence limiting its application in food industries. Various studies have attempted to reduce these limiting factors by modifying extraction methods to achieve a higher yield of protein without denaturation and with higher quality. If they succeed, we can use the easily accessible and non-toxic rice proteins’ functions as well as health benefits in our food; over and above that, we can fulfill the protein requirement of more than half of the world population.

## Figures and Tables

**Table 1:** The essential and non-essential amino acids present in rice protein and composition in g/100 g.

	Amino Acids	Rice Protein (g/100 g)	Reference
Essential amino acid	threonine	2.09–5.06	
	Valine	3.78–6.80	
	Isoleucine	2.69–5.18	
	Leucine	5.30–9.51	
	Lysine	2.2–6.24	
	Histidine	1.19–3.49	[7,15,18,22,24,31]
	Methionine	0.65–3.49	
	Cystine	0.13–3.42	
	Tyrosine	1.33–6.0	
	Phenylalanine	3.5–6.30	
	Methionine + cystine	2.35–3.88	
	Phenylalanine + tyrosine	6.80–10.33	
Non-essential amino acid	Aspartic acid	8.10–10.98	
	Serine	2.96–5.64	
	Glutamic acid	13.36–22.42	
	Glycine	4.21–5.98	[33,34,35,36]
	Alanine	3.69–6.20	
	Arginine	5.30–9.84	
	proline	2.70–14.88	

## Data Availability

Data sharing is not applicable to this article.
